# Cardiac MRI vs. myocardial ^18^F-FDG PET/CT in patients with clinical concern for cardiac sarcoid

**DOI:** 10.1186/1532-429X-17-S1-O30

**Published:** 2015-02-03

**Authors:** Nam Ju Lee, Bongju Lee, Harold Litt

**Affiliations:** 1 Dept. of Radiology, Perelman School of Medicine of the University of Pennsylvania, Philadelphia, PA, USA; 2 Dept. of Mathematics Education, Kyungpook National University, Daegu-si, Korea (the Republic of

## Background

CMR and PET/CT are noninvasive diagnostic tests of choice for cardiac sarcoid, however, there is no standard approach to imaging. Our study compared the presence of delayed enhancement (DE) on CMR with FDG uptake on PET/CT in patients with suspected cardiac sarcoid and evaluated whether the practice of ordering both studies results in improved diagnostic yield.

## Methods

168 patients had both CMR and myocardial PET/CT studies for suspected cardiac sarcoid at a single institution from 11/1/2009 to 9/5/2014. 106 had both studies performed within 3 months; 2 MRIs were nondiagnostic, leaving 104 patients for analysis, mean age 57 (18-80), 33 females and 71 males. The presence of DE on CMR and FDG uptake on PET/CT was compared and stratified by the time between the two exams.

## Results

DE was seen in 79/104 (76%) cases, significantly higher in proportion than FDG uptake in only 31/104 (30%) (p<0.001). Five out of 104 (5%) studies were only PET/CT positive compared to 53/104 (51%) positive only on CMR. When performed within 1 month of CMR, PET/CT increased the yield for disease detection by only 8% (78% vs. 72%) with only one additional case detected with testing over 1 month apart. 5/25 (20%) CMR negative studies were reclassified as positive by PET.

There was more concordance between results (DE+/PET+, DE-/PET-) when performed within 1 month than between 1 and 3 months (51% vs 31%, p=0.06), but no difference in concordance comparing studies performed within 2 weeks and from 2 weeks to 1 month (44% vs. 65%, p>0.1). Conversely, DE+/PET- discordance was more common at 1-3 months than within 1 month (66% vs. 43%, p<0.05). CMR had more positive studies compared to PET at all time intervals between exams (p<0.001), except for 2 weeks-1 month (p>0.1).

## Conclusions

In patients with concern for cardiac sarcoid, DE was present on CMR significantly more often than FDG uptake on PET/CT, even when performed within 3 months of each other. FDG uptake without DE was found in a small minority of patients, and diagnostic yield only increased when studies were performed within 1 month. Concordant studies were more likely within 1 month, with DE+/PET- more common between 1 and 3 months. Therefore, CMR may be useful for baseline evaluation for the presence of disease, and PET/CT may only be useful to assess disease activity (e.g. following treatment) or within 1 month of a negative CMR.

## Funding

N/A.

**Table 1:** Presence of DE and FDG uptake at variable time intervals between CMR and PET/CT studies

PET/MRI	0-2 wks	2wks-1mth	1mth-3mths	total
+/+	15	5	6	26

+/-	2	2	1	5

-/+	26	4	23	53

-/-	8	6	5	20

Total(positive)	52 (43)	17 (11)	35 (30)	104 (84)

